# Contribution of C1485T mutation in the HBx gene to human and murine hepatocarcinogenesis

**DOI:** 10.1038/s41598-017-10570-0

**Published:** 2017-09-05

**Authors:** Satoru Hagiwara, Naoshi Nishida, Ah-Mee Park, Yoriaki Komeda, Toshiharu Sakurai, Tomohiro Watanabe, Masatoshi Kudo

**Affiliations:** 1 Department of Gastroenterology and Hepatology, Kindai University Faculty of Medicine, Osaka-Sayama, Japan; 2 Department of Microbiology, Kindai University Faculty of Medicine, Osaka-Sayama, Japan

## Abstract

Although Hepatitis B virus (HBV) X gene mutations are frequently detected in HBV-related human hepatocellular carcinoma (HCC) patients, causative HBx mutations in the development of HCC have not yet been determined. We herein identified C1485T and C1653T mutations in the HBx gene as independent risk of HCC for HBV through the analysis using serum from chronic hepatitis B patients. We generated transgenic mice expressing wild-type (WT-HBxTg) and mutant (C1485T-HBxTg) HBx to assess the carcinogenic potential of mutated HBx. C1485T-HBxTg mice were more susceptible to diethylnitrosamine-induced hepatocarcinogenesis than WT-HBxTg mice and control non-Tg mice. The promotion of hepatocarcinogenesis in C1485T-HBxTg mice was accompanied by the activation of β-catenin and Jun N-terminal kinase (JNK) signaling pathways as well as the production of reactive oxygen species, whereas the activation of nuclear factor-kappa B in the livers of C1485T-HBxTg mice was attenuated. These results demonstrate that the HBx C1485T mutation contributes to human and murine hepatocarcinogenesis.

## Introduction

Hepatocellular carcinoma (HCC) is the third leading cause of cancer death worldwide and chronic hepatitis B virus (HBV) infection is one of the most important etiologies for the development of HCC^1, 2^. Thus, HBV-related hepatocarcinogenesis is a global health issue. However, the molecular mechanisms responsible for the development of HBV-related HCC have not yet been elucidated in detail.

HBV exerts its oncogenic effects through the integration of its small double-stranded DNA into the host genome of hepatocytes. It is now generally accepted that HBV integration into the host genome plays a critical role in the development of HBV-related HCC^3–5^. Among all portions of HBV genes, the oncogenic role of the HBV-X (HBx) gene in the occurrence of HBV-related hepatocarcinogenesis has been the focus of previous studies because most patients with HBV-related HCC are positive for the expression of HBx at the protein level^6^. The HBx gene encodes a protein of 154 amino acid residues that is composed of an N-terminal negative regulatory/antiapoptotic domain and C-terminal transactivation/proapoptotic domain^7^. Although the exact mechanisms by which the integration of HBx into the host genome causes HCC currently remain unclear, one possible explanation may be the functions of intact HBx as a transcription regulator^8^. It is now generally accepted that the HBx protein positively and negatively regulates the expression of genes associated with apoptosis, inflammation, and oncogenesis and thereby induces hepatocarcinogenesis^9, 10^. Mutations in the HBx gene have also been implicated in HBV-related hepatocarcinogenesis in addition to the role of intact HBx as a transcription regulator. Several studies have demonstrated that HCC-associated HBx mutants more strongly promote oncogenesis than intact HBx^11^. For example, HBx mutants with a C-terminal truncation promote or inhibit cell proliferation in a manner that depends on deletion sites^12^. However, the molecular mechanisms by which the functions of HBx are altered in the presence of HBx mutations and cause the promotion of HBV-related hepatocarcinogenesis have not yet been elucidated in detail. This is also the case for HBx C1653T and C1485T mutations associated with the occurrence of HCC in patients with HBV genotype C in Japan^9, 10^. Therefore, alterations in HBx functions in the presence of HCC-associated mutations need to be examined in more detail in order to clarify the pathogenesis of HBV-related hepatocarcinogenesis.

The present study aimed to examine the characteristics of HBx mutations that increase the risk of the emergence of HCC in chronic hepatitis B (CHB) patients with genotype C and to elucidate the roles of these HBx mutations in the emergence of HCC from damaged livers. We herein demonstrated that HBx C1653T and C1485T mutations are associated with the development of HBV-related hepatocarcinogenesis and also that the latter mutation induces malignant transformation in hepatocytes upon over-expression.

## Results

### Patient characteristics and mutational profile of the HBx gene

In order to examine the causative HBx mutations that lead to the development of HBV-related HCC, we initially attempted to identify the sites of mutations in HBx in CHB patients with or without HCC. For this purpose, CHB patients with or without HCC were retrospectively analyzed in this study. Clinicopathological factors were compared between patients with or without HCC after matching for age, sex, and HBV DNA levels. As shown in Table 1, no significant differences were observed in the positive ratio of HBeAg or serum alanine aminotransferase (ALT) levels between patients with or without HCC. As expected, the ratio of liver cirrhosis (LC) and presence of core promoter mutation were significantly higher in patients with HCC than in those without HCC.

**Table 1:** Characteristics and incidences of various HBx gene mutations in patients with or without HCC.

Variables	non-HCC (n = 40)	HCC (n = 40)	*p*-value
Age (years), median (range)	49.5 (20–77)	53.5 (34–82)	N.S.
Sex (male), n (%)	35 (88%)	35 (88%)	N.S.
HBV-DNA >5 (PCR, log copies/mL), n (%)	23 (58%)	23 (58%)	N.S.
Positive for HBeAg, n (%)	18 (45%)	16 (40%)	0.82
ALT (IU), median (range)	39 (9–246)	44.5 (16–322)	0.69
Pre-core mutation, n (%)	12 (30%)	16 (40%)	0.48
Core promoter mutation, n (%)	23 (58%)	34 (85%)	0.013*
Cirrhosis, n (%)	6 (15%)	23 (58%)	<0.001*
HBx;C1653T mutation	5 (12.5)	15 (37.5)	0.019*
HBx;C1485T mutation	2 (5)	11 (27.5)	0.007*
HBx;C1470A mutation	5 (12.5)	8 (20)	0.55
HBx;C1479A mutation	3 (7.5)	6 (15)	0.48
HBx;C1575G mutation	2 (5)	4 (10)	0.68

HCC, hepatocellular carcinoma; HBeAg, hepatitis B e antigen; PCR, polymerase chain reaction; ALT, alanine aminotransferase; pre-core mutation, a guanine-to-adenine substitution at nucleotide 1896 in the pre-core region; core promoter mutation, an adenine-to-thymine substitution at nucleotide 1762 and guanine-to-adenine substitution at nucleotide 1764 in the core promoter region; N.S., not significant. *Significant difference; *p* < 0.05.

**Figure 1:**
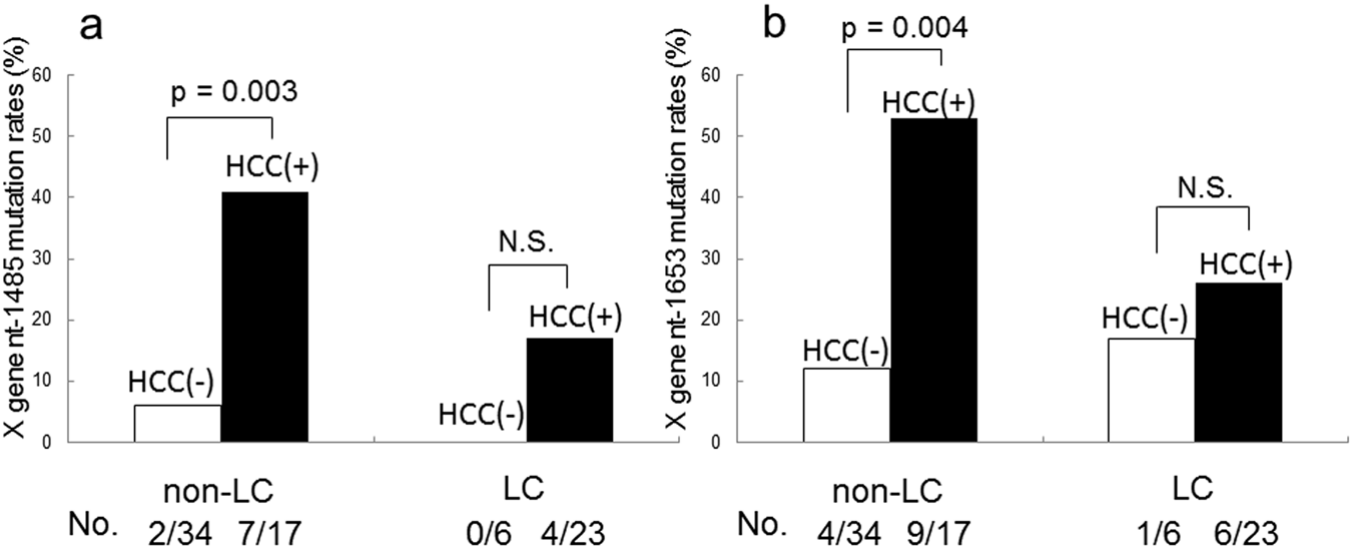
Frequencies of C1485T and C1653T mutation in non-LC and LC cases. Frequencies of C1485T (**a**) and C1653T (**b**) mutations in non-LC and LC cases. White columns represent non-HCC and black gray columns represent HCC patients sample. The numbers in the bottom line mean positive patients number/total patients number.

Since the regions of HBx, the precore, and core promoter in HBV are important for pathogenesis, we sequenced these regions using a specific primer set and examined mutations in these regions. We found that the presence of a double core promoter mutation (A1762T and G1764A) correlated with the development of HCC, as reported previously^13^ (data not shown). We then attempted to identify HBx mutations associated with HBV-related hepatocarcinogenesis and found five different HBx mutations (C1653T, C1485T, C1470A, C1479A, and C1575G) in CHB patients. In the non-HCC group, 6 patients had a single mutation, 4 had 2 mutations, and 1 had 3 mutations in the HBx gene. In the HCC group, 20 patients had a single mutation, 7 had 2 mutations, 2 had 3 mutations, and 1 had 4 mutations in the HBx gene. Among these five HBx mutations, C1485T and C1653T mutations were more frequently detected in HCC than in non-HCC cases (Table 1). The factors identified as significant by a univariate analysis, as listed above, were subjected to a multivariate analysis using a logistic regression model. The presence of the C1485T or C1653T mutation in HBx in combination with LC was identified as an independent factor for the development of HCC (Table 2). Following the identification of the susceptible viral mutations (the C1485T HBx or C1653T HBx mutation) and host factor (the presence of LC) for HBV-related hepatocarcinogenesis in the multivariate analysis, we compared the frequencies of HBx mutations between HCC and non-HCC cases in the context of background liver conditions, i.e. the presence of LC. We compared the frequencies of C1485T and C1653T mutations in LC and non-LC cases. The analysis of patients with non-LC revealed that C1485T and C1653T mutations were more frequent in HCC than in non-HCC cases (p = 0.003 and p = 0.004 for the C1485T and C1653T mutations, respectively; Fig. 1a,b). In addition, there is a trend showing that these mutations were also more frequent even in HCC cases with LC, although a significant difference was not observed in this cohort. Collectively, these results suggest that C1485T and C1653T mutations in HBx contribute more significantly to HBV-related hepatocarcinogenesis in patients without LC than in those with LC where direct role of HBx should be less critical.

**Table 2:** Results of a multivariate analysis using a logistic regression model for assessing risk factors for carcinogenesis.

Variables	Odds ratio	95% CI	*p*-value
Core promoter mutation	3.13	0.88–11.18	0.079
Cirrhosis	11.67	3.37–40.42	<0.001
C1653T mutation	4.70	1.16–18.97	0.030
C1485T mutation	7.75	1.26–47.88	0.027

CI, confidence interval; core promoter mutation, an adenine-to-thymine substitution at nucleotide 1762 and guanine-to-adenine substitution at nucleotide 1764 in the core promoter region. *Significant difference; *p* < 0.05.

### Incidence of HCC in wild-type (WT) and mutant HBx transgenic (Tg) mice

**Figure 2:**
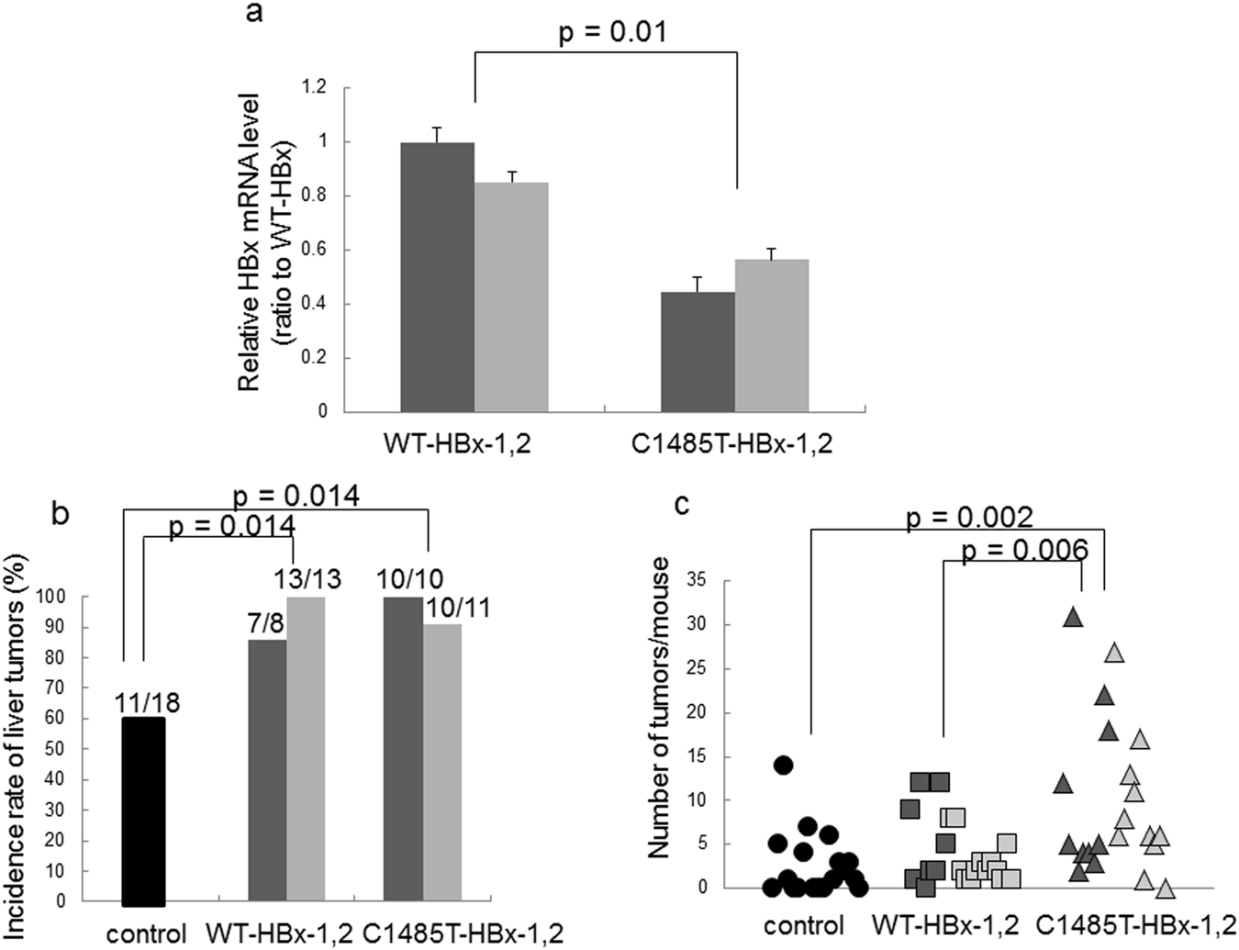
Sensitivity to diethylnitrosamine-induced hepatocarcinogenesis in HBx-transgenic mice. (**a**) HBx mRNA levels in HBx (WT)-transgenic Tg and HBx (C1485T)-Tg mice. Liver were obtained from 6 weeks old male HBx-WT Tg mice and HBx-C1485T-Tg mice. HBx mRNA levels of the liver were determined. The mRNA levels were normalized by β-actin and shown as ratio to one HBx (WT) Tg mice line 1. Data show mean ± standard error (n = 4). Dark gray and light gray columns represent the different lines of the original mice (dark gray Tg line 1, light gray Tg line 2). (**b**) Comparison of the incidence of liver tumors in control non-Tg mice and HBx transgenic mice. Male control non-Tg mice, male HBx-WT Tg mice, and male HBX-C1485T-Tg mice were treated with intraperitoneal injection of diethylnitrosamine (DEN, 25 mg/kg) and then the incidence of liver tumors was determined 8 months after the injection (n = 8–13). Dark and light gray columns represent the different lines of the original Tg mice (dark gray Tg line 1, light gray Tg line 2). (**c**) The number of tumors in each male mouse is represented as a circle in control non-Tg mice, as a square in HBx (WT) mice and as a triangle in HBx (C1485T) mice in the graph. Dark and light gray symbols represent the different lines of the original Tg mouse.

Since the presence of the C1485T mutation showed a higher odds ratio for the development of HCC than the C1653T mutation (Table 2), we focused on the role of C1485T mutation on carcinogenesis and then attempted to directly confirm the oncogenic potential of C1485T-HBx in an *in vivo* experimental model. We created Tg mice overexpressing the HBx C1485T mutation (referred to as C1485T-HBxTg) and WT-HBx (WT-HBxTg). We established two Tg lines overexpressing WT-HBxTg and two Tg lines overexpressing C1485T-HBx. As shown in Fig. 2a, we detected the enhanced expression of HBx mRNA in each Tg mouse, but not in control non-Tg mice.

Previous studies showed that the integrated sites of HBV into host genome play important roles in the development of HCC^4, 5^. We initially tried to determine the integration sites of HBx gene into host genomes. No specific gene loci were identified as integration sites of transgenes in any of WT-HBxTg and C1485T-HBxTg mice (Supplementary Table 1), suggesting that the integration event should result in structural alterations of known cancer-related genes that could enhance oncogenic pathways. Subsequently, we tried to evaluate the development of liver tumor in WT-HBxTg and C1485T-HBx Tg mice. For this purpose, these two Tg mice and control non-Tg WT-C57BL/6 mice were subjected to an injection of diethylnitrosamine (DEN) in order to accelerate hepatocarcinogenesis. As shown in Fig. 2b, the number of male mice that developed liver tumor was significantly higher in the C1485T-HBxTg mouse lines than in control non-Tg mice eight months after the injection of DEN (*p* = 0.014; Fig. 2b). Similarly, each male WT-HBxTg mouse line showed a significantly higher incidence of tumors than control non-Tg mice (*p* = 0.014; Fig. 2b). Although no significant difference was observed in the incidence of liver tumors in male mice among the two C1485T-HBx Tg mouse lines and two WT-HBx Tg mouse lines, there was a significant difference in the number of liver tumors per body between these two lines; the tumor emergence was significantly higher in C1485T-HBxTg mice than in WT-HBxTg mice (*p* = 0.060) as well as control non-Tg mice (*p* = 0.002; Fig. 2c) for male mice. Although the same experiment was performed with female mice, the incidence of liver tumors was low (data not shown). Such low incidence of tumor emergence in female mice may be attributed to the characteristics of DEN-induced hepatocarcinogenesis utilized in this study^14^. Therefore, we used male mice for further analyses.

It might be possible that difference in sensitivity to DEN-induced hepatocarcinogenesis between WT-HBxTg mice and C1485T-HBxTg mice may be caused by different expression levels of HBx between these two lines. Therefore, we performed a quantitative PCR analysis, and found that the HBx mRNA level was not higher, or rather lower, in mice overexpressing C1485T-HBx, which was more susceptible for hepatocarcinogenesis, than in those carrying WT-HBx overexpression (Fig. 2a). Thus, the higher incidence of tumor emergence in C1485T-HBxTg mice could be caused by C1485T mutation rather than the expression levels of HBx. Collectively, these results strongly suggest that hepatocytes overexpressing C1485T-HBx show higher sensitivity to DEN-induced carcinogenesis than those overexpressing intact HBx.

### Increased cell proliferation in livers of C1458T-HBxTg mice

**Figure 3:**
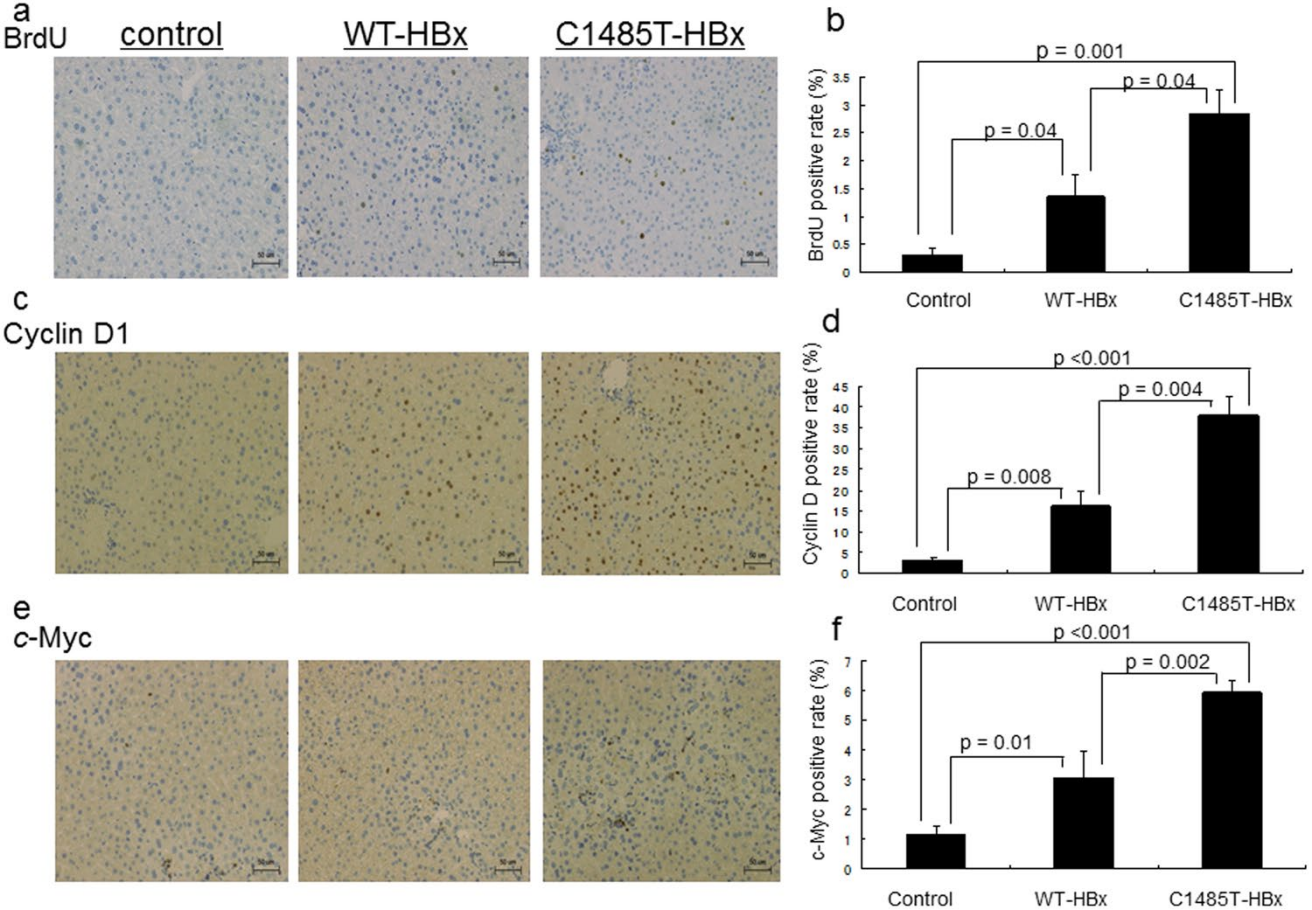
Enhanced proliferation and cell cycle progression in HBx C1485T-transgenic mice. Male control non-Tg mice, male HBx-WT Tg mice (Tg line 1), and male HBX-C1485T-Tg mice (Tg line 1) were treated with intraperitoneal injection of diethylnitrosamine (DEN, 100 mg/kg). Four hours prior sacrifice BrdU was injected intraperitoneally, and mice were sacrificed at 48 hours after DEN injection. Liver sections were prepared and immunohistochemical staining for BrdU (**a**,**b**), cyclin D1 (**c**,**d**), and c-myc (**e**,**f**) was performed. Representative results from at least three independent experiments (n = 4) are shown in panels a, c, and e. Cells positive for each nuclear staining were counted by Image J software and the percentage to total nuclear numbers were shown in graph (**b**,**d**,**f**). Results were shown as mean ± standard error.

In order to confirm the effects of WT- and C1485T-HBx on hepatocyte proliferation, we compared the degree of DNA synthesis in livers after the DEN injection using BrdU staining. As shown in Fig. 3, the greater incorporation of BrdU was observed in the nuclei of hepatocytes in mice overexpressing C1485T-HBx than in those overexpressing WT-HBx and those not expressing HBx (Fig. 3a,b). Moreover, a cell-cycle analysis using cyclin D1 staining also revealed higher numbers of cyclin D1-positive hepatocytes in mice overexpressing C1485T-HBx than in those overexpressing WT-HBx (Fig. 3c,d). Consistent with the results of BrdU and cyclin D1 staining, the expression of the oncogenic protein, c-myc, was significantly enhanced in the hepatocytes of mice overexpressing C1485T-HBx than in those overexpressing WT-HBx and those not expressing HBx (Fig. 3e,f).

### Effects of mutant HBx expression on the activation of cancer-related signaling pathways

**Figure 4:**
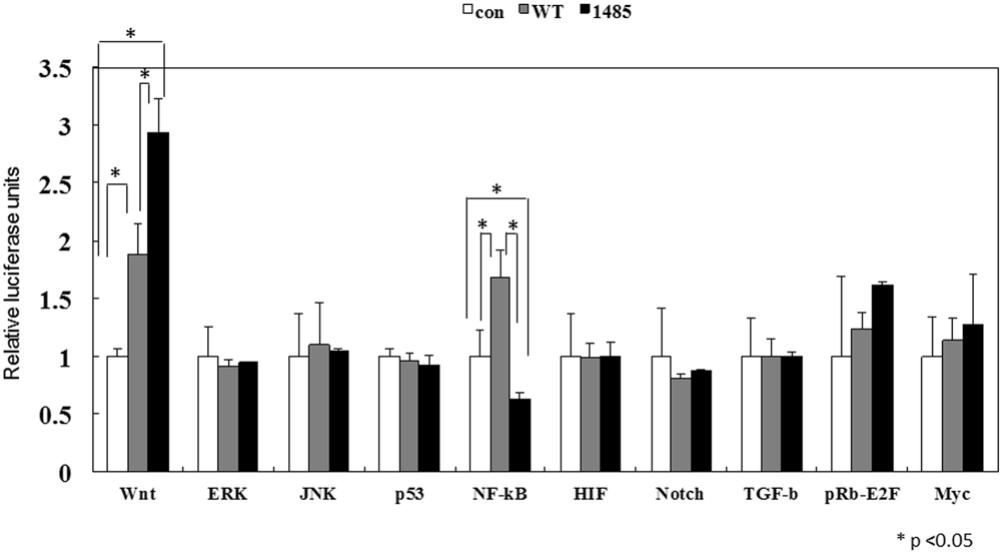
Effect of mutant HBx expression on the activation of cancer-related signaling pathways. Transcription factor activities in HepG2 cells overexpressing HBx were measured by using the Cignal Finder luciferase reporter system. HepG2 cells were transfected with expression vector of wild type HBx (dark gray), C1485T HBx (black), or an empty vector (white). 2 days after transfection, cell lysate were used for this assay. The results are presented as relative luciferase activity mean ± standard error (n = 3).

Following confirmation of the effects of C1485T-HBx on hepatocyte proliferation, as assessed by BrdU and cyclin D1 staining, we attempted to identify the signaling pathways responsible for enhanced hepatocyte proliferation. We analyzed the transcriptional activity of WT- and C1485T-HBx genes using the Cignal Finder Reporter Array, as previously reported^15^ (Fig. 4). Expression vectors containing WT- or C1485T-HBx were transfected into HepG2 cells in order to compare their effects on the activation of several oncogenic pathways (Wnt, extracellular signal-regulated kinase (ERK), Jun N-terminal kinase (JNK), p53, nuclear factor-kappa B (NF-κB), hypoxia induced factor (HIF), Notch, transforming growth factor-β (TGF-β), retinoblastoma protein (pRb)-E2F, and Myc. Transfection with WT- and C1485T-HBx enhanced the transcriptional activity of the Wnt signaling cascade more than the control empty vector. This increase in transcriptional activity was significantly greater in HepG2 cells overexpressing C1485T-HBx than in those overexpressing WT-HBx (p* = *0.007; Fig. 4). In contrast, HepG2 cells overexpressing C1485T-HBx showed weaker NF-κB transcriptional activity than those overexpressing control and WT-HBx (*p* = 0.028 vs. control; *p* < 0.001 vs. wild type-HBx; Fig. 4), whereas the transfection of WT-HBx into HepG2 cells enhanced NF-κB transcriptional activity than that of the control (*p* = 0.012; Fig. 4). The transfection of the WT- or C1485T-HBx gene into HepG2 cells did not alter reporter gene activity regulated by the ERK, JNK, p53, HIF, Notch, TGF-β, pRb-E2F, or Myc signaling pathways, probably because these signaling pathways are constitutively activated in HepG2 cells, as previously described^16^.

### Suppression of NF-κB in livers of C1458T-HBxTg mice

**Figure 5:**
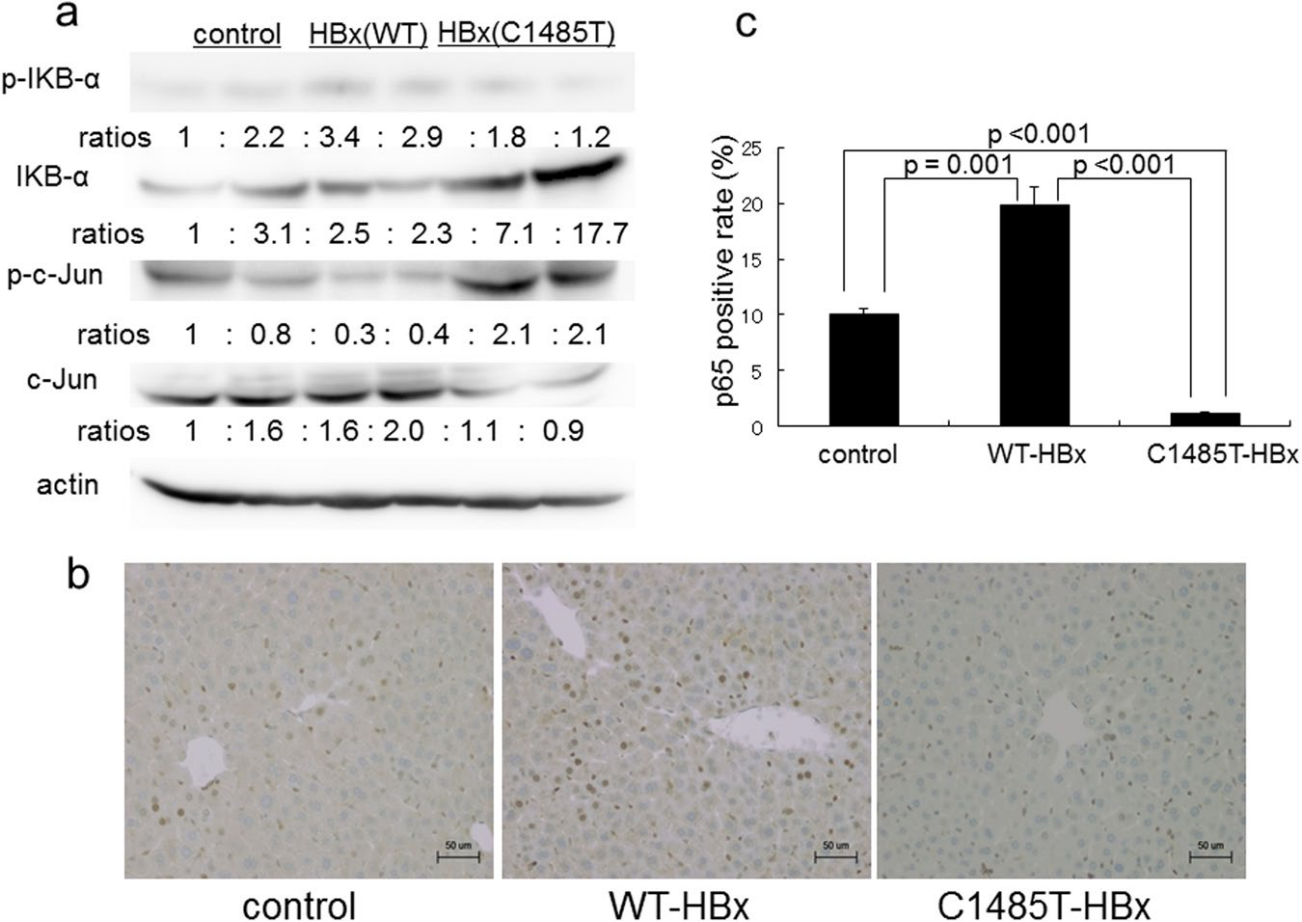
Attenuation of NF-κB activation in the liver of C1485T-HBx transgenic mice. Male control non-Tg mice, male HBx-WT Tg mice (Tg line 1), and male HBX-C1485T-Tg mice (Tg line 1) were treated with intraperitoneal injection of diethylnitrosamine (DEN, 100 mg/kg) and liver tissues were obtained 4 hours later. (**a**) Immunoblot analysis for total and phosphorylated IkB-α and c-jun, with actin as a loading control; each number represents the ratio to the control samples on the lane 1. Lane 1, 2; control non-Tg mice, lane 3, 4; HBx wild type transgenic mice, lane 5, 6; HBx C1485T transgenic mice. (**b**,**c**) Immunohistochemical staining of NF-κB subunit, p65, in the liver. Mice (n = 4, each group) were treated with intraperitoneal injection of DEN as described in (**a**) and liver tissues were obtained 48 hours after DEN injection. Cells positive for nuclear p65 staining were counted by Image J software and the percentage to total nuclear number were shown as mean ± standard error.

The results of the Cignal Finder reporter assay suggested that the presence of C1485T-HBx in hepatocytes inhibited the activation of NF-κB. This result prompted us to investigate the effects of C1485T-HBx on the NF-κB pathway *in vivo*. The phosphorylation and degradation of inhibitor of NF-κB (IκB)-α is an indispensable step for the translocation of NF-κB subunits into the nucleus, followed by the transcription of target genes^17^. We initially analyzed the level of IκB -α phosphorylation (p-IκB -α) in the livers of male control non-Tg and Tg mice carrying WT-HBx and C1485T-HBx four hours after the administration of DEN. As shown in Fig. 5a, the expression of p-IκB -α in the liver was significantly reduced in C1485T-HBxTg mice than in control non-Tg and WT-HBxTg mice. In contrast, the expression of IκB -α in the liver was markedly reduced in WT-HBxTg mice than in C1485T-HBxTg mice. Consistent with the results obtained from immunoblotting, the nuclear expression of p65, a major component of NF-κB subunits, in hepatocytes was attenuated in C1485-HBxTg mice than in WT-HBxTg and control non-Tg mice (Fig. 5b,c). Thus, the results of immunoblotting and tissue staining strongly suggest the suppression of NF-κB activation in the livers of C1458T-HBxTg mice.

We then investigated the activation of JNK signaling pathways because previous studies reported that the up-regulation of the JNK pathway is associated with the down-regulation of NF-κB signaling pathways^18^. The expression of phosphorylated c-Jun in the liver was stronger in C1485T-HBxTg than in WT-HBxTg mice as assessed by immunoblotting (Fig. 5a). Thus, it is conceivable that the presence of C1485T-HBx interferes with NF-κB signaling, the effects of which were accompanied by the activation of c-Jun with the potent ability to accelerate cell proliferation, as reported previously^18^.

**Figure 6:**
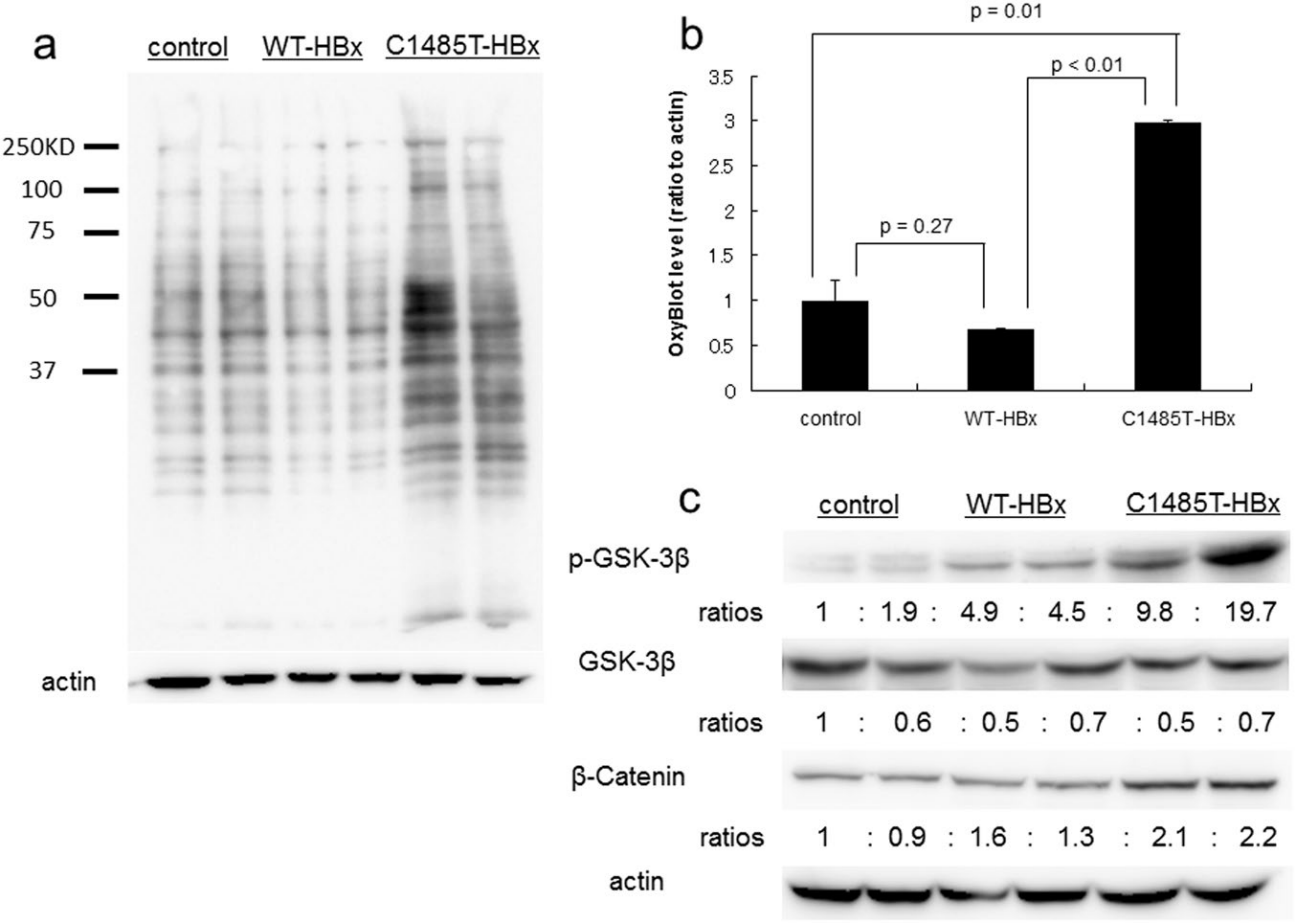
Enhancement of GSK3β and Wnt activation in the liver of C1485T-HBx transgenic mice. Male control non-Tg mice, male HBx-WT Tg mice (Tg line 1), and male HBX-C1485T-Tg mice (Tg line 1) were treated with intraperitoneal injection of diethylnitrosamine (DEN, 100 mg/kg) and liver tissues were obtained 4 hours later. (**a**) Protein oxidation of liver lysate from acute DEN-treated mice was determined by oxyblot analysis kit. Lane 1, 2; control non-Tg mice, lane 3, 4; HBx wild type transgenic mice, lane 5, 6; HBx C1485T transgenic mice. (**b**) The signal intensity of each lane from oxyblot result (**a**) was determined by Image J software. The bar graph presented mean ± standard error (n = 3). (**c**) Immunoblot analysis for total and phosphorylated GSK-β and β-catenin with actin as a loading control; each number represents the ratio to the control samples on the lane 1. Lane 1, 2; control non-Tg mice, lane 3, 4; HBx wild type transgenic mice, lane 5, 6; HBx C1485T transgenic mice.

The production of reactive oxygen species (ROS) in response to the activation of JNK-c-Jun pathways has been implicated in murine hepatocarcinogenesis^19^. Consistent with this finding, oxidized protein levels were significantly higher in C1485T-HBxTg mice than in WT-HBxTg mice, as assessed by OxyBlot^20^ (Fig. 6a,b). Collectively, these results suggest that the promotion of hepatocarcinogenesis induced by the overexpression of HBx-C1485T is accompanied by the enhanced activation of JNK signaling pathways and subsequent production of ROS.

### Activation of Wnt/β-catenin signaling pathways in livers of C1458T-HBxTg mice

We examined the activation status of Wnt signaling pathways because the reporter gene assay showed an increase in activation of the Wnt signaling pathway in WT- and C1485T-HBx-overexpressing cells, particularly in those overexpressing HBx-C1485T. The expression of phosphorylated glycogen synthase kinase-3β (p-GSK3β) and β-catenin, key signaling molecules in the Wnt pathway^21^, was examined by immunoblotting. p-GSK3β expression levels in the liver were higher in C1485T-HBx mice than in control non-Tg and WT-HBxTg mice. Consistent with the results obtained for p-GSK3β, β-catenin levels in the liver were higher in C1485T-HBxTg mice than in WT-HBxTg mice (Fig. 6c). Thus, the promotion of DEN-induced hepatocarcinogenesis observed in C1485T- HBxTg mice was characterized by the enhanced activation of the Wnt signaling pathway combined with attenuated activation of NF-κB signaling pathways.

## Discussion

In the present study, we demonstrated that the C1485T HBx mutation is involved in hepatocarcinogenesis in human and mice. This result is supported by the findings of human studies and experimental models of hepatocarcinogenesis. In human studies utilizing age, sex, and HBV-DNA-matched serum samples obtained from CHB patients, we found that the incidence of HCC was higher in patients bearing C1485T or C1653T HBx mutations than in those bearing wild type HBx. Consistent with the findings of human studies, experimental models of DEN-induced hepatocarcinogenesis revealed that C1485T-HBxTg mice were more susceptible to the development of HCC than WT-HBxTg and non-Tg mice. To the best of our knowledge, this is the first study to conduct an *in vivo* phenotypic analysis on C1485T-HBxTg mice. Collectively, the findings obtained from human and mouse studies support our conclusion that the C1485T mutation in the HBx gene is a susceptible factor for the development of HBV-related hepatocarcinogenesis.

A large number of studies have confirmed the oncogenic roles played by the HBx protein in hepatocarcinogenesis^22^. Since several mutations have been detected in HBx not only in HCC tissues, but also background liver tissue, mutations in HBx have been implicated in the pathogenesis of HBV-related HCC. However, the effects of HBx mutations on the development of HCC currently remain unclear. In the present study, we analyzed the serum of 80 case-matched HBV genotype C-positive patients with or without HCC in order to identify and clarify HBx mutations associated with HBV-related hepatocarcinogenesis. We found that HBV carrying the C1485T mutation in the HBx gene is involved in the pathogenesis of HBV-related HCC. These results are consistent with previous findings by Muroyama *et al*. showing that the C1485T mutation in the HBx gene was associated the development of HBV genotype C-related HCC^10^. Thus, our results together with these findings suggest that the C1485T mutation in HBx might increase the risk of HCC associated with HBV genotype C. Furthermore, the occurrence of the C1485T mutation was markedly higher in HCC patients without LC than in those with LC in our cohort. Therefore, the C1485T mutation in HBx appears to be involved in the development of HCC even in the cases without LC and thereby acts as a potent oncogenic accelerator independent from liver fibrosis.

On the other hand, a previous study showed that HBV sequences derived from tumor and non-tumor tissues were different^23^, indicating that HBx mutations in the serum do not always reflect those in the HCC tissue. In this regards, our preliminary studies show that C1485T mutation was successfully detected in the HCC tissue in 3 of 5 patients bearing such mutation in the serum (data not shown). Previous study also showed that the HBx codon-38 change in human HCC, which is attributed to the presence of C1485T, was detected in corresponding non-tumor tissues, and was consistent with those in serum^10^. These data suggest that detection of the HBxC1485T mutation in the serum, in combination with the analysis of HCC tissue, could be an informative molecular marker to predict the clinical outcome of CHB patients.

The relationship between the C1485T mutation and emergence of HCC has not been confirmed in HBV genotype A infection^24, 25^. Thus, the C1485T mutation in HBx is involved in the pathogenesis of HCC in the presence of HBV genotype C, but not genotype A. In this regard, the infection by HBV genotype C is known to be more strongly associated with severe hepatitis than genotype A of HBV^26^. Since the accumulation of hepatocyte DNA damage is parallel to the severity of hepatitis^27–29^, HBV genotype C infection is regarded as a strong inducer of hepatocyte DNA damage. Thus, the accumulation of DNA damage due to persistent infection with HBV genotype C may act synergistically with the C1485T HBx mutation to promote hepatocarcinogenesis. This is consistent with our phenotypic analysis of C1485T-HBxTg mice in that the spontaneous development of HCC has not been observed in these mice without the administration of DEN (data not shown). Therefore, DEN-induced DNA damage may be a prerequisite for the increased susceptibility of C1485T-HBxTg mice to HCC.

Although several studies have suggested a relationship between mutations in HBx and the development of HCC, specific role of these mutations in hepatocarcinogenesis is still unclear. The relationship observed between HBx mutations and the development of HCC may be an epiphenomenon associated with alternations in the liver microenvironment due to persistent inflammation. Therefore, we established Tg mice carrying C1458T-HBx for the first time and performed a phenotypic analysis. The results obtained from male Tg mice revealed a higher incidence of HCC in C1485T-HBxTg mice than in WT-HBxTg mice following a challenge with DEN. Furthermore, an immunohistochemical analysis revealed that the incorporation of BrdU and expression of cyclin D1 in hepatocytes were stronger in C1485T-HBxTg mice than in WT-HBxTg mice. Thus, the higher incidence of hepatocarcinogenesis in C1485T-HBx Tg mice is linked to the abnormal regulation of the cell cycle and enhanced cell proliferation. Since HBx is a multifunctional protein that not only activates transcriptional transactivation, but also mediates cell growth via proliferation and apoptosis^30^, our results suggest that the C1485T mutation induces hepatocarcinogenesis through enhanced cell proliferation and cell cycle progression. On the other hand, the incidence of liver tumors was low in female mice compared to male mice. Naugler *et al*. showed that DEN-induced hepatocarcinogenesis requires interleukin-6 (IL-6) production by Kupffer cells and that such IL-6 production is negatively regulated by estrogen^14^. Thus, cell proliferation induced by IL-6 could be involved in DEN-induced hepatocarcinogenesis in male mice.

Regarding the mechanisms responsible for enhanced tumorigenesis in C1485T-HBxTg mice, we characterized cancer-related signaling pathways in the presence of C1485T-HBx genes. Reporter gene assays that the transactivation of Wnt signaling pathways was markedly enhanced in HepG2 cells overexpressing C1485T-HBx than in those expressing WT-HBx. Consistently, immunoblotting revealed that the expression of p-GSK3β and β-catenin in the liver was significantly stronger in C1485T-HBxTg mice than in WT-HBxTg mice. Since the activation of the Wnt signaling pathway induces the expression of downstream oncogenic proteins, such as c-myc and cyclin D1, which are overexpressed in the liver of C1485T-HBxTg mice^31^, these results strongly suggest that C1485T-HBx enhances cell cycle progression through the activation of Wnt signaling pathways. Given the fact that WT-HBx also induces HBV-related carcinogenesis through the activation of the Wnt/β-catenin signaling pathways^32^, our results indicate that the presence of the C1485T HBx mutation further enhances hepatocarcinogenesis by augmenting Wnt/β-catenin signaling pathways.

The suppression of NF-κB activation was significantly greater in HepG2 cells overexpressing C1485T-HBx than in those overexpressing WT-HBx. Consistent with this result, the activation of NF-κB was markedly suppressed in the livers of C1485T-HBxTg mice, as assessed by the expression of phospho-IκB α and degradation of IκB α. Furthermore, the nuclear translocation of p65, a major NF-κB subunit, in hepatocytes was more strongly inhibited in C1485T-HBxTg mice than in WT-HBxTg mice. Thus, the emergence of HCC caused by C1485T-HBx is also attributed to the suppression of NF-κB. Such suppression of NF-κB in the presence of C1485T- mutation is in contrast to previous findings showing that HBx induces malignant transformation through the inhibition of hepatocyte apoptosis and promotion of angiogenesis in an NF-κΒ-dependent manner^33, 34^. This discrepancy in the status of NF-κB activation may be partially explained by the types of cells that show the activation of NF-κB. Maeda. *et al*. demonstrated that hepatocyte-specific IKKβ-deficient mice exhibited a marked increase in hepatocarcinogenesis caused by DEN^35^. In addition, the enhancement in hepatocarcinogenesis observed in this hepatocyte-specific NF-κB -deficient mice was accompanied by an increase in the accumulation of ROS^35^. Consistent with the findings reported by Maeda *et al*., enhancements in DEN-induced hepatocarcinogenesis in C1485T-HBxTg mice were characterized by significant increase of ROS with the suppression of NF-κB activation in the liver, which are involved in hepatocarcinogenesis in C1485T-HBxTg mice treated with DEN.

The increase of phosphorylated c-Jun is another characteristic associated with the development of HCC in the presence of C1485T-HBx in mice model. It should be noted, however, that no difference was detected in JNK activation in HepG2 cells overexpressing WT-HBx gene and C1485T-HBx gene in the reporter gene assay (Fig. 4). The discrepancy of JNK activation between *in vitro* and in *vivo* experiments can be partially explained by the fact that JNK pathway is constitutively activated in HepG2 cells, which makes difficult to detect a significant difference in cell line study^16^.

Regarding the mechanisms responsible for the activation of c-Jun in mice model, we speculate that the accumulation of ROS induced by the suppression of NF-κB plays a role in the activation of JNK signaling pathways. The accumulation of ROS has been reported to induce the oxidative inhibition of mitogen-activated protein kinase (MAPK) phosphatases, which are enzymes responsible for terminating the activation of JNK, and then cause the persistent activation of JNK signaling^36^. Since JNK signaling pathways are regarded as an important contributor to hepatocyte proliferation and HCC development^37–39^, the activation of these pathways may be involved in enhanced hepatocarcinogenesis observed in C1485T-HBxTg mice. In addition, activation of JNK is reportedly contributed to the expansion and proliferation of stem-progenitor cells^40^; the C1485T-HBxTg mice showed an increase of CD133^+^ stem-progenitor cells compared to WT-HBxTg mice after treated with DEN (data not shown). Based on these findings, the accumulation of ROS followed by the activation of JNK may also be contributed to C1485T-HBx –dependent hepatocarcinogenesis.

In conclusion, we identified a novel mutation in the HBx gene that accelerates hepatocarcinogenesis. In human studies, we found that the C1485T HBx mutation was more frequent in the sera of patients with HCC than in those without HCC. In experimental models of hepatocarcinogenesis, C1485T-HBxTg mice were more susceptible to the development of HCC than WT-HBxTg mice. The development of HCC in the presence of the C1485T-HBx mutation is associated with the enhanced activation of the Wnt and JNK signaling pathways, decreased activation of NF-κB signaling pathways, and accumulation of ROS. We consider the results of the present study to be of significance in terms of basic research and clinical perspectives. From a basic research standpoint, C1485T-HBxTg mice are a useful tool for the study of the molecular mechanisms underlying HBx-related hepatocarcinogenesis. Our results suggest, from a clinical viewpoint, that a screening of C1485T-HBx mutation in serum might represent a promising approach for predicting the emergence of HCC for CHB, particularly in patients carrying HBV genotype C.

## Methods

### Patient characteristics

In order to analyze the mutational profile of HBV, we selected 80 out of 185 consecutive hepatitis B surface antigen-positive patients who visited Kindai University Hospital between January 1998 and December 2005. Among these patients, 40 harbored HCC, while 40 had never had this condition. Age, sex, and HBV DNA levels were matched between HCC-positive and -negative groups. Refer to the Supplementary Materials and Methods for more details.

The study protocol conformed to the ethical guidelines of the 1975 Declaration of Helsinki and was approved by the Institutional Review Board of Kindai University Faculty of Medicine. Written informed consent was obtained from all patients recruited in the study. All animals received humane care and the study protocol complied with the institution’s guidelines.

### HBV-DNA status of patients

Samples from all patients were examined for the following serological markers: the hepatitis B surface antigen, hepatitis B e antigen, anti-HBe antibody, HBV DNA level, and HBV genotype. α-Fetoprotein, the lens culinaris A-reactive fraction of α-fetoprotein, and des-γ-carboxy prothrombin were also examined. Regarding the genotype of HBV, all patients had genotype C infection. Further details on the methods used to assess these parameters are described in the Supplementary Materials and Methods. Patient characteristics according to the presence or absence of HCC are listed in Table 1.

### Reporter assays on HB-transfected HepG2 cells for the detection of altered cellular signaling

We conducted reporter assays to clarify differences in transcriptional activities between WT and mutant HBx. The HBx gene was amplified from the serum DNA of CHB patients. The primers for WT-HBx were: 5′-ttCTCGAGATGGCTGCTCGGGTGTGC-3′ (HBx forward) and 5′-ttGATATCTCAGACGGAGGTGAAAAAG-3′ (HBx reverse), and amplified products were cloned into the pEBMulti-Hyg expression vector (Wako Pure Chemical). Regarding the C1485T mutation in HBx, we used the same primer set as that used for the construction of HBx-mutant Tg mice (see below). Transfection of the plasmid (control, WT-HBx, C1485T-HBx, empty vector) into HepG2 cells was performed using Fuge-6 (Roche) following the manufacturer’s instructions. The expression of the HBx protein was confirmed by immunocytochemistry using an anti-HBx antibody (BioVendor, Modrice, Czech Republic). These cells were used in subsequent reporter assays.

The reporter assay for the cancer pathway was performed using the Cignal Finder Cancer 10-Pathway Reporter Array (SA Biosciences, Fredrick, MD) according to the manufacturer’s instructions. HepG2 cells transfected with the pEBMulti expression vector containing WT- or C1485T-HBx were seeded onto a 96-well plate. Forty-eight hours after transfection, cell lysates were added to Luciferase Assay Reagent II, and firefly luciferase activity was measured. An empty construct was used as a negative control for the normalization of transcriptional activity.

### DEN-initiated tumorigenesis in transgenic mice

Transgenic mice expressing WT- and C1485T-HBx were generated using C57/BL6 mice (Charles River Laboratories Japan, Yokohama, Japan). Details on the construction are provided in the Supplementary Materials and Methods. The animal experiments were approved by the institutional Animal Care and Use Committee of Kindai University and performed in accordance with the institutional guidelines.

Two-week-old Tg and non-Tg mice were intraperitoneally injected with DEN at 25 mg/kg body weight (Sigma-Aldrich, St Louis, MO). Eight months after the injection, mice were euthanized with an overdose of pentobarbital (200 mg/kg) and cut open for photography and tissue harvesting. Livers were excised, weighed, and examined for macroscopic lesions. According to standard methods, livers were fixed in 10% neutral buffered formalin (Wako Pure Chemical, Osaka, Japan), dehydrated, embedded in paraffin, sectioned serially at 5 µm, and stained with hematoxylin and eosin. Tumorous and non-tumorous liver tissues were stored immediately at −80 °C. Identification of integration sites of transgene in the C57/BL6 mice was described in Supplementary Materials and Methods.

### Western blots and analysis of protein oxidation in hepatocytes

DEN (100 mg/kg body weight) was intraperitoneally injected into 4-week-old Tg and non-Tg mice 4 hours before sacrifice. The liver tissue of Tg mice was homogenized with CelLytic-MT Mammalian Tissue Lysis/Extraction reagent (Sigma-Aldrich, St. Louis, MO) containing a protease inhibitor (Complete; Roche Diagnostics, Mannheim, Germany) and phosphatase inhibitor cocktail (Nacalai Tesque, Kyoto, Japan). Tissue lysates were electrophoresed on a reducing sodium dodecyl sulfate (SDS)-polyacrylamide gel and electroblotted onto a polyvinylidene difluoride (PVDF) membrane. The membrane was blocked with 5% skimmed milk and incubated with anti-phospho-ser32-IκB -α, anti-c-Jun, anti-phospho-ser73-c-Jun, anti-signal transducer and activator of transcription (STAT) 3, anti-phospho-ser727-STAT3, anti-GSK3β, anti-phospho-ser9-GSK3β, anti-β-Catenin (Cell Signaling Technology, Inc. Danvers, MA), and anti-β-actin (Sigma-Aldrich, St. Louis, MO) antibodies. Protein levels were detected using horseradish-peroxidase-linked secondary antibodies and the ECL-plus System (GE Healthcare, Buckinghamshire, UK). In order to evaluate signal intensity, Western blot image data were quantified using ImageJ software (NIH, Bethesda, MD).

Protein oxidation was assessed using the OxyBlot Protein Oxidation Detection Kit (Millipore Bioscience Research Reagents, Temecula, CA). Twenty micrograms of protein was reacted with dinitrophenylhydrazine for 15 min, followed by neutralization with a solution containing glycerol and 2-mercaptoethanol, resolved using 10% SDS-polyacrylamide gel electrophoresis, and transferred to a PVDF membrane using a semidry transfer system (BioRad). Membranes were then blocked with phosphate buffer saline Tween (0.05% Tween-20) containing 0.1% bovine serum albumin at room temperature for 1 hour, and incubated with a rabbit anti-2,4-dinitrophenol antibody (1:150) overnight at 4 °C. The secondary antibody incubation was performed using a horseradish peroxidase-conjugated anti-rabbit IgG (1:300) at room temperature for 1 hour. Immunoreactivity was visualized by enhanced chemiluminescence using ECL plus reagents (GE Healthcare, Piscataway, NJ).

### Immunohistochemistry

DEN (100 mg/kg body weight) was intraperitoneally injected into 4-week-old Tg and non-Tg mice. Four or 48 hours after the injection, mice were sacrificed and subjected to immunohistochemical analyses. In *in vivo* bromodeoxyuridine (BrdU) labeling, BrdU (50 mg/kg, Wako) was injected 4 hours prior to sacrifice. The liver was isolated, fixed in 20% formalin for 18 h, and stained using the BrdU *In-Situ* Detection kit (BD Pharmingen, San Diego, CA). Immunohistochemistry was performed with a Histofine SAB-PO Kit (Nichirei Biosciences, Tokyo, Japan). Anti-cyclin D1 (Cell Signaling; Beverly, MA), anti-c-myc (Santa Cruz Biotechnology, Santa Cruz, CA), and anti-NF-κB p65 (Cell Signaling) antibodies were used as primary antibodies.

### Statistical analysis

In order to compare differences between patients with or without HCC, categorical and continuous variables were analyzed using Fisher’s exact test and the Mann-Whitney U test, respectively. Factors with a significant difference detected in univariate analyses were subjected to multivariate analyses using a logistic regression analysis model. A p of <0.05 was considered to be significant. All analyses were performed with SPSS software (version 11.5; SPSS Inc., Chicago, IL).

## Electronic supplementary material

Supplementary Information
