# Supplementary material for: Contribution of C1485T mutation in the HBx gene to human and murine hepatocarcinogenesis
Source: Sci Rep. 2017 Sep 5;7:10440. doi: 10.1038/s41598-017-10570-0 (PMC5585302; doi:10.1038/s41598-017-10570-0)

Contribution of C1485T mutation in the HBx gene to human and murine hepatocarcinogenesis

Satoru Hagiwara[1], Naoshi Nishida[1], Ah-Mee Park[2], Yoriaki Komeda[1], Toshiharu Sakurai[1], Tomohiro Watanabe[1], and Masatoshi Kudo[1]

[1]Department of Gastroenterology and Hepatology and [2]Department of Microbiology, Kindai University Faculty of Medicine, Osaka-Sayama, Japan

Table of contents

Supplementary materials and methods

References

Supplementary Table 1

Supplementary Figure 5a

Supplementary Figure 6c

Supplementary Materials and Methods

Diagnosis of HCC

A diagnosis of HCC was based on histological findings in 33 cases. In the remaining 7 cases, the diagnosis was made according to reliable clinical criteria [1,2] that fulfilled the following conditions: a clinical background compatible with HCC; the typical imaging findings for this condition; and increased levels of HCC-related tumor markers, in particular a serum α-fetoprotein (AFP) level of >400 ng/mL, serum des-γ-carboxy prothrombin level of ≥100 mAU/mL, or AFP-L3 level of >15%. Typical imaging findings for HCC included: a mosaic pattern with a halo on B-mode ultrasonography; hypervascularity on angiography; and a high-density mass on arterial-phase dynamic computed tomography (CT) and low-density mass on portal-phase dynamic CT. When typical findings for HCC were not obtained through dynamic CT or angiography, CT during hepatic arteriography and CT during arterial portography or T1- and T2-weighted imaging obtained from superparamagnetic iron oxide-enhanced magnetic resonance imaging were performed.

Status of HBV-DNA patients

The hepatitis B surface antigen, hepatitis B e antigen, and anti-hepatitis B e antibody were assessed using an enzyme immunoassay (Sysmex, Kobe, Japan). Serum AFP was measured with a chemiluminescent enzyme immunoassay (Lumipulse AFP-N; Fujirebio Inc., Tokyo, Japan). The Lens culinaris agglutinin A-reactive fraction of AFP (AFP-L3)

was measured using a liquid-phase binding assay (LBA Wako AFP-L3; Wako Pure Chemical Industries, Ltd., Osaka, Japan). AFP-L3 levels were expressed as a percentage of the total AFP concentration: (AFP-L3 concentration/total AFP concentration) × 100. The serum des-γ-carboxy prothrombin level was measured using an electrochemiluminescence immunoassay (PicolumiPIVKA II; Eisai Co., Ltd., Tokyo, Japan). Serum HBV DNA was quantified using a real-time polymerase chain reaction (PCR) assay (Amplicor HBV Monitor; Roche Diagnostics, Tokyo, Japan). HBV genotypes were assessed in sera by PCR of the S-gene region, and the restriction fragment length polymorphism pattern was analyzed as described by Mizokami et al. [3].

Identification of mutations present in the HBx gene, precore region, and core promoter

In order to identify the mutations harbored in the HBx gene, precore region, and core promoter, HBV DNA was extracted from serum samples using a NucleoSpin Blood Kit (Macherey-Nagel GmbH & Co., KG, Germany). The HBx gene was amplified using the following primers: 5′-GCC AAC TTA CAA GGC CTT TCT-3′ (forward, position 1101–1121) and 5′-TTC ATC AAC TCA CCC CAA CTC-3′ (reverse, position 2100–2080), and the products obtained were purified using a Wizard SV Gel and PCR Clean-Up System (Promega Co., Madison, WI). Sequences were elucidated with ABI3100 (Applied Biosystems, Foster City, CA) using the nested primers 5′-AGC CGG TCT GGA GCA AAA CTT-3′ (forward, position 1303–1323) and 5′-AGT AAC TCC ACA GAA GCT CCA-3′ (reverse, position 1947–1927). The presence of a mutation

resulting from a guanine-to-adenine substitution at nucleotide 1896 in the precore region and a double mutation in the core promoter region (adenine-to-thymine substitution at nucleotide 1762 and guanine-to-adenine substitution at nucleotide 1764) were assessed.

Mutations in the HBx gene were identified through comparisons with the standard sequence deposited in GenBank under accession number AB206817. When sequencing revealed the heterogeneity of the HBV gene in a patient, the major sequence was adopted for genotyping.

HBxTg mice harboring the wild-type or mutant HBx gene

Four different HBxTg mouse lines were used in our studies: WT-HBxTg-1 and WT-HBxTg-2 were 2 independent mouse lines that carried the WT-HBx genome; C1485T-HBxTg-1 and C1485T-HBxTg -2 were 2 independent mouse lines that carried the mutant-type HBx genome. All mouse lines were in the B6 genetic background. HBxTg mice were generated based on a previously reported method [4]. Briefly, a 1150-base pair (bp) EcoRI-XhoI HBV DNA fragment was cloned by PCR using HBV genomic DNA (genotype C) obtained from the serum of a hepatitis B patient. The primers for the WT-HBx gene fragment were: 5′-AACCCTAATAAAACCAAACGT-3′ (forward) and 5′-TCGAATAGAGGAAAAGAAGTC-3′ (reverse). Regarding HBx with the C1485T mutation, each primer contained a mutated base (underlined) and were as follows: 5′-GGGCCTCTATCGTCCTCTT-3′ (1485 mutation forward) and 5′-AAGAGGACGATAGAGGCCC-3′ (1485 mutation reverse). After purification, PCR fragments were digested with EcoRI and XhoI (New England BioLabs, Beverly, MA) at

37°C, purified, and ligated into the pcDNA3.1 vector using T4 DNA ligase (Epicentre, Madison, WI). The recombinant vector plasmids containing the WT or mutant HBx gene were digested with EcoRI and XhoI. DNA fragments were then microinjected into c57Bl/6J oocytes.

Reverse transcription-PCR (RT-PCR) was performed for the detection and quantification of the HBx transgene. Total RNA (1 µg) extracted from the liver using TRIzol (Invitrogen, Carlsbad, CA) was reverse-transcribed with a High Capacity RNA-to-cDNA kit (Applied Biosystems, Foster City, CA). The resultant cDNA was amplified using Quick Taq HS DyeMix (Toyobo) and loaded onto a 1.5% agarose gel containing ethidium bromide. The HBx gene was amplified using the PCR primers 5′-ATGGCTGCTCGGGTGTGC-3′ (5′ primer) and 5′-TCAGACGGAGGTGAAAAAG-3′ (3′ primer) to produce a 465-bp product. Denaturing was performed at 95°C for 30 s, annealing at 56°C for 45 s, and polymerase reactions at 72°C for 45 s (33 cycles). GAPDH mRNA levels were also monitored using the primers 5′-CATCACCATCTTCCAGGAGC-3′ (5′ primer) and 5′-GGAAGGCCATGCCAGTGAGC-3′ (3′ primer) as an internal control.

Assessment of HBx mRNA levels by quantitative PCR

Total RNA was extracted from WT-HBxTg and C1485T-HBxTg mouse livers using an RNeasy mini kit (Qiagen). RNA samples (500 ng each) were reverse-transcribed using the High Capacity RNA-to-cDNA kit (Applied Biosystems, Foster City, CA). Quantitative PCR (qPCR) was performed using the Power SYBR Green PCR Master

Mix (ABI) and ABI Step One Real-Time PCR system, with b-actin as a reference control. The sequences of the sense and antisense primers used for amplification were: HBX, 5′-TGTCAACGACCGACCTTGAG-3′ and 5′-CCCCAACTCCTCCCAGTCTT-3′; b-actin, 5′-GACGTTGACATCCGTAAAGA-3′ and 5′-GCAGTAATCTCCTTCTGCAT-3′.

The determination of integration sites of the transgenes

For the determination of integration sites of the transgenes into the C57/BL6 mice genome, we used Universal GenomeWalker™ 2.0 kit (Clontech Laboratories, Inc. Mountain View, CA) according to the manufacture’s protocol. Briefly, the genomic DNA from transgenic mice was digested with 4 kinds of restriction enzyme, *Dra* I, *Eco*R V, *Pvu* II, and *Hpa* I). Each batch of digested genomic DNA was ligated separately to the Genome Walker adopter, followed by the first amplification of nested-PCR using outer adopter primer and outer primer specific for HBx gene. The mixture of primary PCR product was subjected to the 2nd PCR using inner adopter primer and inner HBX specific primer. Each nested PCR product was subject to the direct sequencing by Sanger method, which should contain breakpoints between host and HBx genome**.**

Primer sequences used for the amplification of PCR product containing breakpoints are listed below.

Primers	Sequences
Outer primers specific for HBx gene	aacaagctgctaggagttccgcagtatg
	catactgcggaactcctagcagcttgtt
Inner primers specific for HBx gene	tccaattacatatcccatgaagttaagg
	aattggtctgttcaccagcaccatgcaa
Outer adopter primer	gtaatacgactcactatagggc
Inner adopter primer	actatagggcacgcgtggt

References

[1] Torzilli G et al. Accurate preoperative evaluation of liver mass lesions without fine-needle biopsy. Hepatology **30**, 889-893 (1999).

[2] Kudo M. Imaging diagnosis of hepatocellular carcinoma and premalignant/borderline lesions. Semin Liver Dis. **19**, 297-309 (1999).

[3] Mizokami M et al. Hepatitis B virus genotype assignment using restriction fragment length polymorphism patterns. FEBS Lett. **450**, 66-71 (1999).

[4] Kim CM, Koike K, Saito I, Miyamura T, Jay G. HBx gene of hepatitis B virus induces liver cancer in transgenic mice. Nature **351**, 317-320 (1991).

Supplementary Table 1

Integrations of transgenes in the C57BL/6J mice carrying HBx with C1485T mutation and wild-HBx genes

Lines	Integration of transgenes[1]			
	Chromosome	Expect value	Percentage of identity	Sequence ID
C1485T-HBxTg				
Line 1	chromosome 8	9e-156	93% (364 / 392 bp)	AC076974.33
Line 2	chromosome X	8e-68	99% (145 / 146 bp)	NC_000086.7 [2]
WT-HBxTg				
Line 1	chromosome 16	3e-140	99% (278 / 282 bp)	NC_000082.6 [3]
Line 2	N.D		N.D	N.D

C1485T-HBxTg: transgenic mice carrying HBx with C1485T mutation.

WT-HBxTg: transgenic mice carrying wild type-HBx.

N.D; not determined.

[1] The sequences of the mouse genome adjacent to HBx gene were determined using Universal Genome Walker™ 2.0 (Materials and Methos). Chromosome with integration, expected value, percentage of identity, and sequence ID in database were determined using the Basic Local Alignment Search Tool (BLAST)-NCBI.

[2] Location: 1069222 bp at 5' side of the 60S ribosomal protein L30-like, and 426711 bp at 3' side of the CPX chromosomal region candidate gene 1 protein homolog.

[3] Location: 242878 bp at 5' side of the enteropeptidase isoform 1 precursor, and 666012 bp at 3' side of uncharacterized protein 1700066C05Rik.

Figure 5a

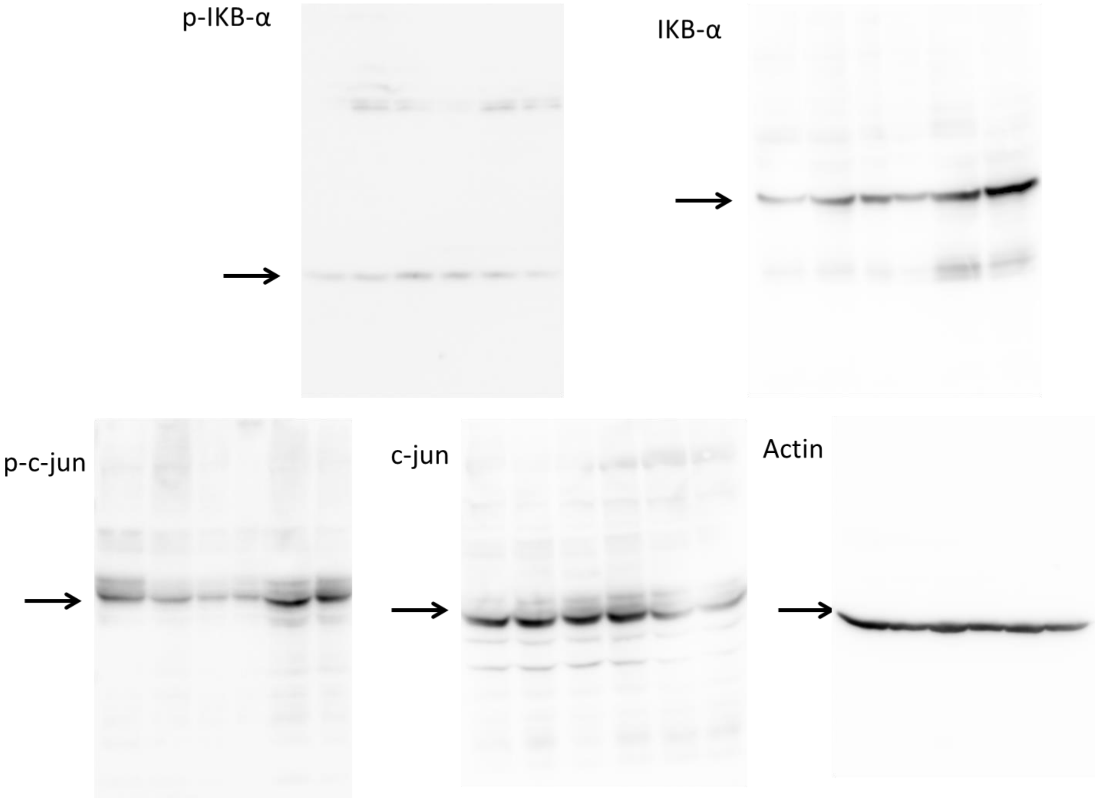

Figure 6c

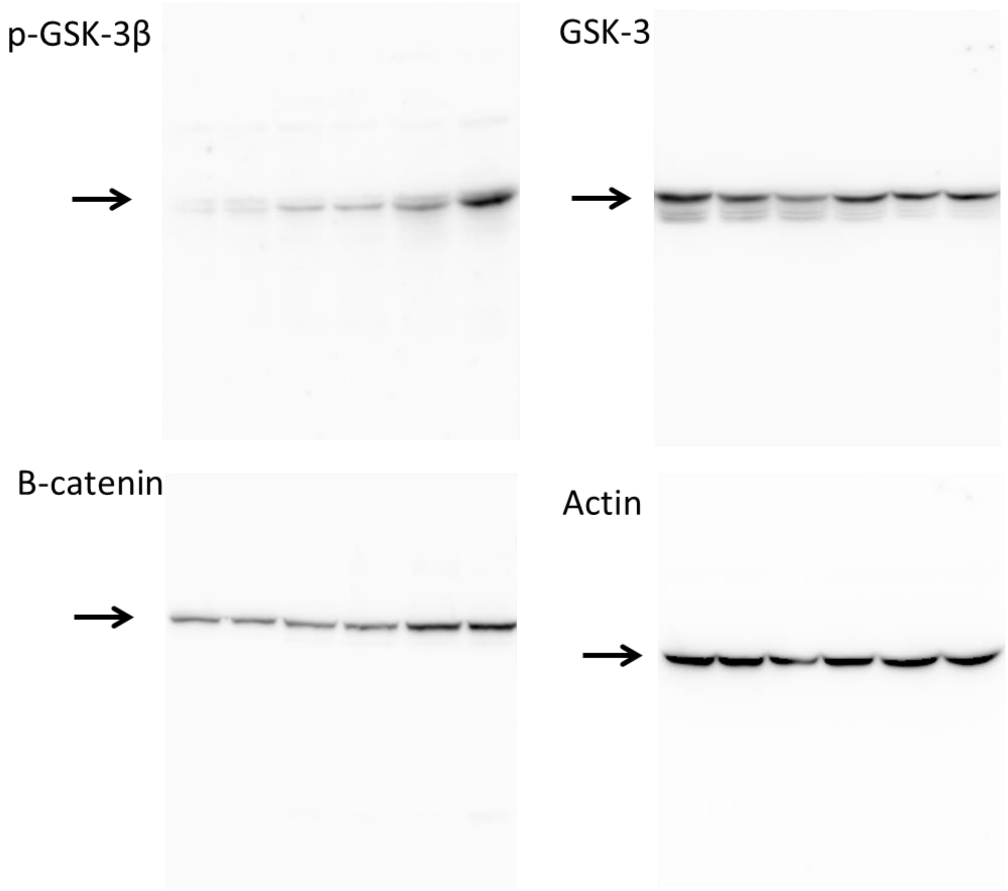

Supplement: Supplementary file 1 — Supplementary Information [file 41598_2017_10570_MOESM1_ESM.pdf]
